# Comparing the effect of two educational interventions on mothers’ awareness, attitude, and self-efficacy regarding sexual health care of educable intellectually disabled adolescent girls: a cluster randomized control trial

**DOI:** 10.1186/s12978-021-01112-z

**Published:** 2021-03-02

**Authors:** Shadi Goli, Mahnaz Noroozi, Mehrdad Salehi

**Affiliations:** 1 Nursing and Midwifery Sciences Development Research Center, Najafabad Branch, Islamic Azad University, Najafabad, Iran; 2 Department of Midwifery and Reproductive Health, School of Nursing and Midwifery, Isfahan University of Medical Sciences, Isfahan, Iran; 3 Medical School, Isfahan University of Medical Sciences, Isfahan, Iran

**Keywords:** Intellectual disability, Adolescent, Sexual health, Training, Awareness, Attitude, Self-efficacy

## Abstract

**Background:**

Sexual problems of intellectually disabled adolescents are associated with their inability to understand sexuality. Given the important role of parents in forming the attitude of their adolescents towards sexuality, the present study aimed to compare the effect of two educational interventions on mothers’ awareness, attitude and self-efficacy regarding sexual health care of intellectually disabled adolescent girls.

**Materials and methods:**

This cluster randomized control trial was conducted in six intellectually disabled adolescent education centers in Isfahan, Iran in 2018. The centers were randomly assigned to intervention groups (group training and training through booklet) and control group. Mothers of educable intellectually disabled adolescent girls (n = 81) were entered into the three groups using convenience sampling and their awareness, attitude and self-efficacy regarding sexual health care of adolescent girls were assessed using questionnaires before and after the educational intervention. Data were analyzed using descriptive and inferential statistical methods.

**Results:**

The mean score of mothers’ awareness, attitude and self-efficacy in each of the “group training”, “training through booklet group” and “control group” was significantly different after the intervention compared to before the intervention (p < 0.05). The mean score of mothers’ awareness and self-efficacy after the intervention in the “group training” was higher than the “control group” and “training through booklet group” (p < 0.001). The mean score of mothers’ awareness and self-efficacy after the intervention in the “training through booklet group” was higher than in “control group” (p = 0.005, p = 0.02). Also, after the intervention, the mean score of mothers’ attitude in the “group training” was higher than the “control group” and the “training through booklet group” (p < 0.001), but there was no significant statistical difference between the mean score of mothers’ attitude in “control group” and “training through booklet group” (p > 0.05).

**Conclusion:**

Implementation of the group training intervention for mothers of intellectually disabled adolescent girls in comparison with training through booklet was associated with a greater increase in their awareness, attitude and self-efficacy regarding sexual health care of adolescent girls. Therefore, group training is suggested as a suitable way to educate mothers about sexual health care of intellectually disabled adolescent girls.

*Trial registration* IRCT, IRCT20160224026756N5. Registered 22 June 2018, https://en.irct.ir/user/trial/31704/view.

## Plain English summary

Intellectual disability refers to concomitant impairment in adaptive behavior that manifest during developmental years and at less than 18 years of age. Training parents plays an important role in helping them in the care of their adolescent’s sexual health. The purpose of this study was to compare the effect of two educational interventions on mothers’ awareness, attitude and self-efficacy about the sexual health care of intellectually disabled adolescent girls. This study was conducted in six intellectually disabled adolescent education centers in Isfahan, Iran in 2018. Mothers of educable intellectually disabled adolescent girls were entered into the three groups (group training, training through booklet and control group) and their awareness, attitude and self-efficacy regarding sexual health care of adolescent girls were assessed using questionnaires before and after the educational intervention. The results showed that implementation of the group training in comparison with training through booklet was associated with a greater increase in mothers’ awareness, attitude and self-efficacy about the sexual health care of intellectually disabled adolescent girls. Therefore, group training is suggested as a suitable way to educate mothers about sexual health care of intellectually disabled adolescent girls.

## Background

Intellectual disability refers to concomitant impairment in adaptive behavior that manifest during developmental years and at less than 18 years of age [1]. Causal factors in the creation of intellectual disability include genetic disorders, prenatal exposure to infections and toxins, perinatal trauma, acquired disorders and socio-cultural factors. Approximately 85% of intellectually disabled (ID) people fall into the mild (educable) category [2]. These individuals with intelligence quotient (IQ) range from 50 to 55 to 70 advances up to sixth grade elementary school in education and with limited social support; they can be guided towards social compliance and acquisition of independence [3]. Adolescent girls with intellectual disability do not learn how to control their sexual desires and express it in accordance with human laws and in a societal manner [4,5]. For this reason, they exhibit inappropriate behaviors such as masturbation and exhibitionism in public can lead to their vulnerability to sexual abuse and harassment and consequences like unwanted pregnancies, sexually transmitted infections (STIs) and HIV/AIDS [6,7]. Numerous studies have shown that id adolescent girls are abused by family members, caregivers, close relatives, and other community members [8–12].

Since studies suggested that sexual problems of id adolescents are rooted in their inability to understand sexuality, unawareness and inability of parents, caregivers and teachers multidimensional interventions must be considered [13–15]. Parents play an important role in forming the attitude of id adolescents toward puberty and sexuality ^[[[[16]]]]^. Yıldız and Cavkaytar in a study showed that sexual training program can cause positive changes in mothers’ attitude towards sexual education and improve their understanding of social support [17]. The results of other studies showed that training parents with id children plays an essential role in enhancing their awareness and self-efficacy, changing their attitudes and behaviors about sexual education and talking to their child about sexuality [18,19]. Regarding the difference of socio-cultural and religious context of Iran with other countries and the taboo of talking about sexual issues [20,21], limited studies have been done in this area [4–6,20]. Therefore, it is necessary to perform studies to determine the effect of training parents about sexual health care of id adolescent girls. Sexual health education programs should be tailored to gender, age, level of knowledge, and socio-cultural background. Given the possibility of providing training in different ways, choosing the right method has an important role in the success of the training [22]. One of the training methods is group training. Group training is a way of stimulating thinking that challenges beliefs and attitudes and teaches individual skills. In this way, if the learners are ready to participate in the discussion or the subject is understandable to them, training can gain an acceptable success [23]. Another method of training is through booklet which usually can be used when there is not sufficient number of trainers [24]. Given the special role of mothers in caring for their adolescent girls, sexual health and regarding so far no study has been done to compare the effect of different training methods on mothers’ awareness, attitude and self-efficacy about sexual health care of id adolescent girls, so, the present study was conducted to compare the effect of two educational interventions on mothers’ awareness, attitude and self-efficacy about the sexual health care of id adolescent girls.

## Methods

CONSORT guidelines were adhered for reporting of this trial.

### Study design

This study (as a part of expanded mixed methods study [25]) was a cluster randomized control trial with three groups (intervention 1, intervention 2 and control). The population of this study includes mothers of id adolescent girls who referred to six id adolescents’ training centers covered by Welfare Organization and Special Education Organization of Isfahan city, Isfahan province, Iran from June to August 2018. There were an appropriate number of id adolescent girls and their mothers with the same socio-economic status who referred to these centers. Based on the confidence interval of 95%, a test power of 80% (using G-power software), d = 2.9 for awareness, d = 11.3 for attitude, d = 5 for self-efficacy and considering a sample loss rate of 10%, sample size of 27 was estimated for each group.

### The inclusion and exclusion criteria

The inclusion criteria were mothers who had not previously completed adolescent’s sexual health education or child sexual abuse prevention courses; mothers were not educated in the field of medicine, allied medical sciences, and psychology; and mothers who could read and write. Exclusion criteria were reluctant to continue collaborating in any stage of the study and lack of full participation in the training sessions held in the group training (failure to receive 50% of the intervention).

### Procedures

**Fig. 1:**
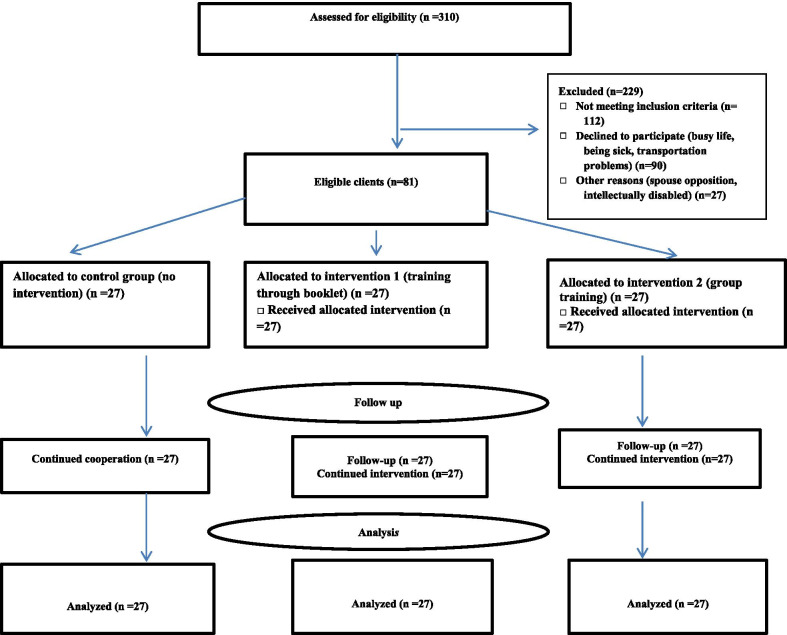
Trial flowchart

In the present study, for preventing relationships and interactions between participants about the educational interventions, from six id adolescents’ training centers, two were randomly selected for “group training”, two for “training through booklet” and two for “control group” by first author (SG). Samples were selected in each id adolescents’ training center using the convenience sampling. In the centers, by examining 310 records of adolescent girls, their mothers’ were called and the purpose of the study was given to them and they were invited to participate in the study. Ninety mothers refused to participate in the study. Then, since out of 139 mothers who referred to the six centers, 81 eligible mothers were enrolled and after obtaining written consent, they were entered in the study (Fig. 1). For the participants in “group training” (n = 27), four training sessions of one-week interval was hold for each subgroup (four subgroups of 6–7 persons). In “group training”, the researcher provided the training content in three stages of introduction, presentation and conclusion. To avoid boring sessions, the presentation of the content was done by a lecture for 45–50 min according to the research objectives, and by discussing, question and answer, mothers were encouraged to activity and their experiences was shared. The aim of this question and answer was to increase mothers’ awareness, change her attitude and enhance self-efficacy in the field of sexual health care of id adolescent girls. During the “group training” sessions, educational items were expressed in a way that mothers consider the issue of their daughter’s sexual health care to be important and know themselves to be able to care for and protect their daughter’s sexual health. Mothers were also asked to provide examples to describe their successful experiences about taking care of their daughter’s sexual health and to discuss this with each other in this subject. Also, by encouraging and emphasizing their individual capabilities in taking care of their daughter’s sexual health, it was tried to increase their self-efficacy. At the end of the session, the main concepts were reviewed, the contents presented were linked together, summarized, and questions were answered. In the case of mothers who were assigned to the group of “training through booklet”, after delivering the booklet, they were reminded that they would be answered by telephone if they had a question. The contents of the booklet were the same as those taught in “group training” sessions. In formulating the training booklet, the training content was also presented in a way that mothers, consider the issue of their daughter’s sexual health care to be important and know they are able to take care for and protect their adolescent’s sexual health. In other words, the training content of the booklet was designed to encourage mothers to apply, the tips contained in it to take care of their daughter's sexual health in a practical and accessible way. Educational content included puberty, adolescence and its changes; female genitalia; menstruation and common menstrual problems in id adolescent girls; common concerns of parents during adolescence period in their daughter; sexual desires and behaviors of id adolescent girls; purpose and necessity of sexual health education; appropriate opportunities for teaching sexual health to the adolescent; use the correct names for different parts of the body; privacy, private and public parts of the body; private and public places; body rights and private conversations; duties of parents in preventing sexual harassment and abuse in adolescents; sexual harassment and abuse in cyberspace; the rules for using the Internet and cyberspace; unwanted pregnancies and STIs; inappropriate sexual behaviors (masturbation, exhibitionism, etc.) in adolescents and how to control these behaviors. In the present study, there was no intervention in the control group and participants only completed the questionnaires in two stages. In this regard, a training booklet was given to the participants in control group after the intervention.

### Primary outcomes

The primary outcomes included mothers’ awareness, attitude and self-efficacy about the sexual health care of their id adolescent girls that were measured by the researcher-made questionnaires (awareness and attitude questionnaires, as well as general self-efficacy questionnaire [23]. These questionnaires were completed by the participants at two stages (before the intervention and one month after the intervention) in id adolescents’ training centers. The first part of the questionnaire included demographic characteristics (age, occupation and education level; number of children; age, occupation and education level of spouse; and age and level of education of id adolescent). The mothers’ awareness questionnaire consisted of 23 questions and the questions had three choices of “True, False and Don't Know”. In assessing the score of awareness, one point was given to the person for each correct answer. Minimum awareness score was zero and maximum was 23. The mothers’ attitude questionnaire consisted of 18 questions and was given one to five points based on a 5-point Likert scale with choices of “strongly agree, agree, neutral, disagree and strongly disagree”. Minimum attitude score was one and maximum score was 90. Standard general self-efficacy questionnaire consisted of 10 four-point questions “not at all correct, hardly correct, almost correct, and completely correct” and was scored on a four point Likert scale from one to four. The minimum self-efficacy score was one and the maximum was 40 [26]. Content validity method was used to determine the validity of the questionnaires of awareness and attitude assessment, and test–retest was used to confirm their reliability. Questionnaires reliability was confirmed by Pearson’s correlation coefficient of 0.91.

### Statistical analysis

Statistical analysis of the present study was conducted using SPSS software (version 23). The one-way ANOVA and Fisher’s exact test were used for assessing the consistency of the three groups in terms of socio-demographic characteristics. To compare mean score of mothers’ awareness, attitude and self-efficacy in each group paired t-test was used before and after the intervention. To compare the mean score of mothers’ awareness, attitude and self-efficacy between the three groups one-way ANOVA was used before and after the intervention. Also, Post hoc LSD test were used for comparison of means difference between two groups after the intervention. The significance level for statistical tests was considered less than 0.05 (P < 0.05).

### Ethical considerations

This study was registered in the registry for clinical trials (IRCT20160224026756N5). The Ethics Committee of Isfahan University of Medical Sciences approved the protocol of this study (code number: IR.MUI. Rec.1395.3.281). Written informed consent, confidentiality, anonymity, and the right of leaving the research at any desired time were preserved.

## Results

The results showed that the mean age of mothers and their spouses, level of education and job of mothers and their spouses, mean number of their children, mean age and level of education of their id adolescent girl were not significantly different between the three groups (p > 0.05) (Table 1).

**Table 1:** Comparison of socio-demographic characteristics of the participants between the three groups

Variable	Group training N (%)	Training through booklet N (%)	Control group N (%)	p-value*
**Age (years)**				0.63
25–34	4 (15)	3 (11)	2 (8)	
35–44	11 (40)	12 (44)	12 (45)	
45–54	11 (41)	11 (41)	11 (40)	
≥ 55	1 (4)	1 (4)	2 (7)	
**Spouse age (years)**				0.74
35–44	4 (15)	3 (11)	2 (7)	
45–54	11 (40)	13 (48)	10 (38)	
≥ 55	12 (45)	11 (41)	15 (55)	
**Number of children**				0.23
1	3 (11)	2 (7)	1 (4)	
2	9 (34)	13 (48)	10 (37)	
≥ 3	15 (55)	12 (45)	16 (59)	
**Educational level**				0.17
Elementary	1 (4)	0 (0)	0 (0)	
Intermediate	9 (34)	12 (44)	9 (33)	
High school and diploma	13 (47)	9 (34)	14 (52)	
Academic	4 (15)	6 (22)	4 (15)	
**Job**				0.31
Employee	4 (15)	5 (19)	6 (22)	
Worker	2 (7)	0 (0)	0 (0)	
Housewife	20 (74)	17 (63)	16 (59)	
Other	1 (4)	3 (18)	5 (19)	
**Spouse educational level**				0.29
Elementary	2 (7)	1 (4)	1 (4)	
Intermediate	10 (37)	8 (29)	11 (41)	
High school and diploma	11 (41)	14 (52)	10 (37)	
Academic	4 (15)	4 (15)	5 (18)	
**Spouse job**				0.44
Unemployed	2 (7)	1 (4)	2 (7)	
Worker	4 (15)	7 (26)	8 (30)	
Employee	11 (41)	9 (33)	10 (37)	
Self employee	6 (22)	9 (33)	4 (15)	
Other	4 (15)	1 (4)	3 (11)	
**Age of id adolescent girl**				0.23
11–15	10 (37)	14 (52)	12 (44.5)	
16–20	17 (63)	13 (48)	15 (55.5)	
**Educational level of id adolescent girl**				0.23
Elementary	8 (29)	3 (11)	9 (34)	
Primary high school	4 (15)	18 (67)	14 (52)	
Secondary high school	14 (52)	6 (22)	2 (7)	
Diploma	1 (4)	0 (0)	2 (7)	

*Id* intellectually disabled

^*^P < 0.05 was considered significant

### Primary outcomes

The results showed that the mean score of mothers’ awareness, attitude and self-efficacy in each of the “group training”, “training through booklet group” and “control group” after the intervention was significantly different from that before the intervention (p < 0.001) (Tables 2, 3, 4). The mean score of mothers’ awareness, attitude and self-efficacy before the intervention was not significantly different between the three groups (p > 0.05), but one month after the intervention, the mean score of mothers’ awareness, attitude and self-efficacy showed a significant statistical difference between the three groups (p < 0.001) (Table 5). In this regard, one month after the intervention, the mean score of mothers' awareness, attitude and self-efficacy was significantly different in the “group training” compared with the “training through booklet group” and “control group” (p < 0.001). Based on the results, the mean score of mothers' awareness and self-efficacy was significantly different in the “training through booklet group” compared with the “control group” (p = 0.005, p = 0.02), but the mean score of mothers' attitude was not significantly different in the “training through booklet group” compared with the “control group” (p > 0.05) (Table 6).

**Table 2:** Comparison of the mean score of mothers’ awareness, attitude and self-efficacy before and one month after the intervention in the group training

Variable	Before the intervention		One month after the intervention		Statistical test results	
	Mean	SD	Mean	SD	F	p
Awareness	9.81	9.05	17.07	1.59	1.49	< 0.001
Attitude	58.81	12.72	82.11	4.51	1.69	< 0.001
Self-efficacy	22.92	5.01	33.03	4.09	0.81	< 0.001

**Table 3:** Comparison of the mean score of mothers’ awareness, attitude and self-efficacy before and one month after the intervention in the training through booklet

Variable	Before the intervention		One month after the intervention		Statistical test results	
	Mean	SD	Mean	SD	F	p
Awareness	12.18	3.41	15.14	2.08	− 9.92	< 0.001
Attitude	61.74	9.80	69.96	7.71	− 6.95	< 0.001
Self-efficacy	22.70	6.03	28.70	5.25	− 3.87	< 0.001

**Table 4:** Comparison of the mean score of mothers’ awareness, attitude and self-efficacy before and one month after the intervention in the control group

Variable	Before the intervention		One month after the intervention		Statistical test results	
	Mean	SD	Mean	SD	F	p
Awareness	11.44	3.76	13.25	2.62	− 6.67	< 0.001
Attitude	64.22	8.90	68.14	8.01	− 9.69	< 0.001
Self-efficacy	24.00	5.28	25.66	4.25	− 7.03	< 0.001

**Table 5:** Comparison of the mean score of mothers’ awareness, attitude and self-efficacy one month after the intervention between the three groups

Variable	Group training		Training through booklet		Control		Statistical test results	
	Mean	SD	Mean	SD	Mean	SD	F	p
Awareness	17.07	1.59	15.14	2.08	13.25	2.62	21.37	< 0.001
Attitude	82.11	4.51	69.96	7.71	68.14	8.01	32.39	< 0.001
Self-efficacy	33.03	4.09	28.70	5.25	25.66	4.25	17.78	< 0.001

**Table 6:** Pairwise comparison of the mean score of mothers’ awareness, attitude and self-efficacy one month after the intervention

Variable	Control Group and Group training		Training through booklet & control group		Group training & Training through booklet	
	Mean difference (95% CI)	p	Mean difference (95% CI)	p	Mean difference (95% CI)	p
Awareness	3.81 (2.5–6.2)	< 0.001	1.88 (0.3–59.18)	0.005	1.92 (0.2–91.93)	< 0.001
Attitude	13.96 (10.17–41.51)	< 0.001	1.81 (2.6–48.11)	0.40	12.14 (8.15–69.60)	< 0.001
Self-efficacy	7.37 (5.9–8.65)	< 0.001	3.03 (0.5–42.64)	0.023	4.33 (1.6–75.90)	< 0.001

*CI* confidence interval

## Discussion

The present study aimed to compare the effect of two educational interventions on mothers’ awareness, attitude and self-efficacy regarding sexual health care of id adolescent girls. The results showed that the mean score of mothers' awareness, attitude and self-efficacy after the intervention in each of "group training" and "training through booklet group" was significantly higher than before the intervention. In this regard, the results of Kok and Akyuz,s study about the effect of group training on parent’s awareness and self-efficacy in managing id adolescent girls' sexual development showed that parent^’^s awareness and self-efficacy was increased after training [27]. Also, a study showed that significant changes were made in parental attitudes after educational intervention through laptop and they gained the confidence and skills needed to provide their disabled child with sex education [18]. The results of the present study showed that the mean score of mothers' awareness, attitude and self-efficacy in the "control group" was significantly higher than before the intervention. It seems that mothers’ exposure to sexual issues related with id adolescent girls (follow the completion of the questionnaires) has faced them with concerns and questions that they have tried to gain information through relatives, friends, and other sources during the study. The results of the present study showed that one month after the intervention, the mean score of mothers' awareness, attitude and self-efficacy was higher in the “group training” than the “training through booklet group” and “control group “. Rashid and Hosseini Nazarlou showed that group training was effective in enhancing parent’s awareness and feeling of competence regarding sexual education of their child [28]. Kardan et al. believed that the motivating role of the teacher in group training can provide the conditions for understanding new content [29]. Shahraki Sanavi et al. in their study concluded that to change attitudes, group training is needed and individual and self-taught methods are not effective [30]. It seems that in the present study, due to the presence of the researcher in the “group training” and creating an opportunity for question, answer and discussing about id adolescent girls, sexual health, not only learning is done better, but also the researcher has been able to eliminate the mothers, wrong beliefs and create a positive attitude towards the issue of sexuality of id adolescent girls. Also, in “group training”, due to the researcher's effective role in encouraging mothers and emphasizing their individual abilities in taking care of their daughters' sexual health, their self-efficacy increased more than training through booklets. Babayanzad Ahari et al. concluded that parents required training to enhance their knowledge and skills to improve their communication with their adolescents about sexuality issues through culture-appropriate educational programs [31]. Furthermore, in a study, the major barriers about sexual health education for adolescent daughters identified by the mothers were their own insufficient knowledge about sexual issues, embarrassment surrounding discussions of this issue with their daughters, fear of the arrogance and curiosity of girls, and a lack of skills for effective communication [32]. Thus, based on the results of present study, “group training” can help mothers overcome barriers and empower them to take care of their daughter’s sexual health. In the present study, the content of booklet was designed in such a way that mothers while becoming aware of their id adolescent girl’s sexual health issues, know to be able to care for and protect their daughters' sexual health. Therefore, mothers' awareness and self-efficacy regarding sexual health care of id adolescent girls was increased in “training through booklet group". But, similar to the results of other studies [33], it seems that “training through booklet”; one may not be able to positively affect mothers' beliefs and attitudes.

### Practical implications

Findings from the present study can be useful in designing and improving the educational methods available in the health system and ministry of education for educating mothers with id adolescent girls. In this regard, by using the “group training”, mothers find a positive attitude towards the sexuality of the id adolescent girls and can be empowered to solve the sexual health problems of their daughters and to deal properly with unexpected events. Also, using educational booklets can increase mothers' awareness and self-efficacy about caring for the sexual health of their id adolescent girls.

### Strengths and limitations

One of the strengths of this study is the implementation of educational intervention on mothers’ awareness, attitude, and self-efficacy regarding sexual health care of educable id adolescent girls, for the first time. One of the limitations of this study is the sensitivity and taboo of sexual issues and the feeling of shame about addressing these issues that can influence information gathering. Mothers' attitudes toward sexual problems of id adolescent girls were also affected by varying degrees of social, religious, moral, and legal norms that were out of control. In addition, mothers' individual differences in learning and motivation were factors associated with the effect of education and it was not possible to control this variable. Mothers' familial, social, and economic problems also affected how to answer the questions which could not be controlled. Since the effect of clustering had not been considered while calculating sample size, this could have affected the results of the research.

## Conclusion

The findings of this study showed that “group training” compared to “training through booklet”, has a more positive effect on mothers' awareness, attitude, and self-efficacy in the care of sexual health of educable id adolescent girls and empowers them to manage sexual behaviors of their id adolescent girls and deal with unexpected events. Therefore, designing and applying “group training” is recommended in mothers' educational programs about sexual health care of id adolescent girls.

## Data Availability

The datasets generated and/or analysed during the current research are not publicly available as individual privacy could be compromised but are available from the corresponding author on reasonable request.

## Abbreviations

IQ: Intelligence quotient
ID: Intellectually disabled
STIs: Sexually transmitted infections
HIV: Human immunodeficiency virus
AIDS: Acquired immune deficiency syndrome
